# Free will beyond confusion: what matters for criminal law, forensic practice, and everyday life

**DOI:** 10.3389/fpsyg.2026.1811791

**Published:** 2026-07-17

**Authors:** Stephan Schleim

**Affiliations:** 1 Theory and History of Psychology, Faculty of Behavioral and Social Sciences, Heymans Institute for Psychological Research, University of Groningen, Groningen, Netherlands; 2 Stephan Schleim Philosophy and Psychology, Amersfoort, Netherlands

**Keywords:** conscious control, criminal law, free will, neuroethics, responsibility, volition

## Introduction

**Figure 1:**
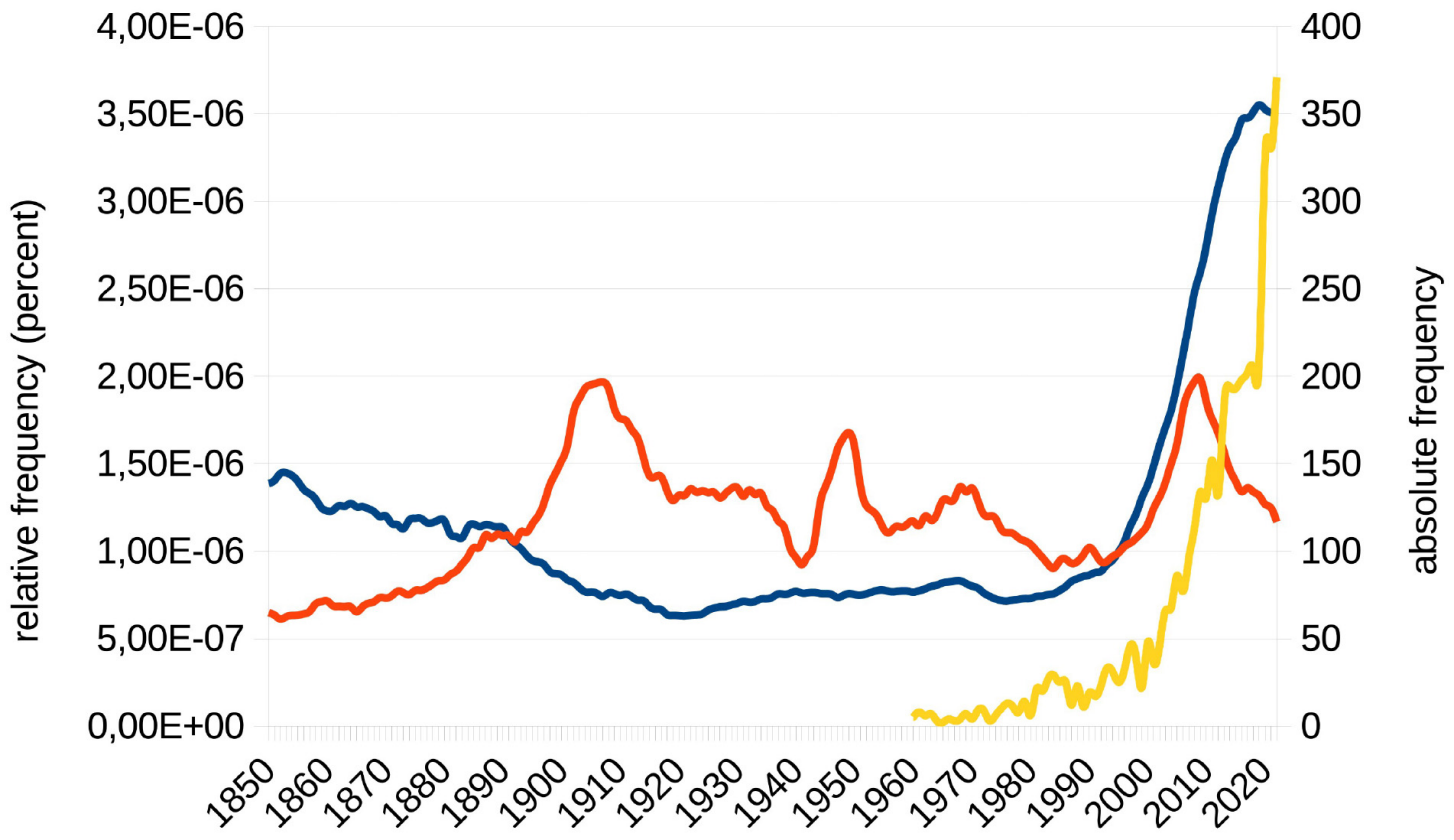
The graph shows the relative frequency of the term “free will” in English books (blue line) and “Willensfreiheit” in German books (red line, both left scale) and the absolute number of papers on “free will” in the Web of Science (yellow line, right scale, topic search). While this suggests a fluctuation of the debate in German-speaking countries with peaks in the early 1900s, around 1950, and 2010, the relative increase in English books and the absolute increase in scholarly papers suggest increasing relevance of the debate since the early 2000s. Data source: Google Ngram, Web of Science.

The topic of free will has received much attention in psychology, neuroscience, philosophy and law since the 1990s, the “Decade of the Brain” (Figure 1). What is less well-known is that there was already an intensive debate on the topic in German-speaking countries in the 19th century, including the calls for a new criminal law. For example, the German-Swiss scientist Carl Vogt (1817–1895), who was well-known to the public, wrote about the issue:

“Free will does not exist and with it no responsibility such as morality and the criminal justice system [...] want to impose on us. At no moment are we masters of ourselves, of our reason, of our intellectual powers, any more than we are masters of whether our kidneys should secrete or not secrete. The organism cannot control itself; it is controlled by the law of its material composition. What we think in a moment is the result of the current mood, the current composition of our brain [...]” [(Vogt, 1852), p. 445f].

Vogt, who is also known for the comparison that the brain produces thoughts like the kidney produces urine, considered brain activation to be determined by natural laws. This would leave no room for free will. The idea that we are not “masters of ourselves” anticipated Sigmund Freud's (1856–1939) very similar formulation by around 75 years (Freud, 1917; Schleim, 2012). From today's perspective, we would call Vogt a “hard determinist,” that is, someone who considers the world (or at least the nervous system) to be completely determined and thus free will to be impossible. From the two binary variables, (in-)determinism and (no) free will, neurophilosophers have distinguished four positions that can be put in a 2×2 matrix (Table 1): besides the already mentioned hard determinist these are the libertarian, who assumes indeterminism, and the compatibilist (Roskies, 2006; Walter, 2009; Schleim, 2024). Representatives of the latter two positions defend free will, but define it differently. For libertarians, it is a metaphysical will that determines our naturally undetermined decisions; and compatibilists, by contrast, see freedom of will in precisely the right kind of determination, namely through our own desires, beliefs and conscious deliberation. The theoretically conceivable fourth position that both determinism and free will are ruled out (i.e., the lower right cell in Table 1) is hardly taken, probably because it has no added value.

**Table 1:** The 2×2 matrix for the two binary variables free will and determinism in the free will debate.

Determinism?			
		**Yes**	**No**
**Free will?**	**Yes**	Compatibilism	Libertarianism
	**No**	Hard determinism	–

Note that the position denying free will and determinism (lower right) is hardly taken.

My aim here is not to provide yet another summary of the philosophical debate. I would just like to briefly note three thoughts about the positions mentioned: first, no one can say exactly what a “will” is. The eminent analytic philosopher Gilbert Ryle (1900–1976) already noted that “volition” (Latin *voluntas* = will) is an “artificial concept” that people do not normally use in everyday language; its use overlaps with the questions of whether people act voluntarily or intentionally and whether they are responsible for their actions (Ryle, 1949/2009). We will pursue these further in the following section. Second, as Table 1 exemplifies, much of the free will debate revolves around the argument about whether the world is deterministic. Philosophers have interpreted this in terms of whether one could have decided and acted differently under *exactly* the same circumstances (Dennett, 2003). In computer science, an algorithm is considered deterministic if it leads to the same output with the same input, given the same internal state. In quantum physics, it is still disputed whether states of the world are sometimes only indeterminate or truly random (Wüthrich, 2011). At the same time, there is a discussion about the possible connection between quantum physics and consciousness (de Barros and Montemayor, 2019). I consider these questions to be practically unproductive: neither do we—in a constantly changing environment and with a complex, dynamic brain with around 86 billion nerve cells and many other cells—ever find ourselves in *exactly* the same conditions twice, nor would truly random decisions represent an interesting kind of free will. Even a vending machine will not produce the same output with the same input if, for example, the power fails. Nor do we have the opportunity to start the whole universe over again to find out whether it leads to *exactly* the same state at time t. Thirdly, the compatibilist simply shifts the problem to another level: if decisions are free when they are made consciously by oneself in accordance with one's preferences, this says nothing about the origin of these preferences (Sie and Wouters, 2010). For example, how free is someone in their dealings with other people if they have grown up in an environment in which the inferiority of certain ethnic groups of people has been taught? Or as the philosopher Arthur Schopenhauer famously said: you can do what you want, but you cannot want what you want (Schopenhauer, 1841/1978).

This is not the place to discuss these questions further (Schleim, 2024). Instead, in Section 2, I want to show that we do not have to answer them for our practice of law and in everyday life. In this context, it is interesting to reinterpret frequently cited neuroscientific experiments, which I do in Section 3. The paper will conclude with a brief summary and outlook.

## Free will in criminal law, forensic practice, and real life

In my opinion, the confusion in the discussion of free will arises from the fact that a thing called “will” is assumed to be free or unfree (Schleim, 2020a). However, more recent philosophical discussions show that the meaning of “will” is by no means clear and that it is more of a moral concept (Hieronymi, 2022). This is not a problem for legal and forensic practice, however, as the law does not presuppose such an entity: both German and US criminal law—as probably also that of many other countries—assume that people are usually, at least in a minimal sense, rational beings who are responsible for their actions. For example, under German law, anyone over the age of 14 is criminally responsible unless, for example, there is a pathological impairment of insight or control (Roxin and Greco, 2020; Schleim, 2026). Can someone distinguish between right and wrong and act on this insight? US-American criminal law requires a causal connection between the criminal act (actus reus) and criminal mind (mens rea) (Dressler, 2015; Morse, 2007). If, say, Adam wants to kill Bob with poison, Adam cannot be prosecuted for murder if Bob happens to die in a traffic accident, even after ingesting the poison but before the poison acts—and in spite of Adam's intention and (otherwise sufficient) action to kill him. There can be no murder case, because someone already killed in a traffic accident cannot be murdered as well; the causal link will not be completed, because of another and earlier cause of death (Dressler, 2015). However, if Adam succeeds in killing Bob, he might be legally exculpated if he meets one of the specific reasons for excuse, such as duress or a specific mental disorder (Dressler, 2015; Morse, 2007).

In criminal proceedings, judges and experts therefore do not need to discuss whether someone had “free will,” but whether there is a legally defined excuse. For example, even in the case of a clear pathological finding such as a brain tumor, the extent to which this impairs the perception, thinking, decision-making and, in particular, the conscious control of a defendant would have to be assessed, where “control” is understood as goal selection, maintenance, and execution.^1^ This is because not every person with a brain tumor, even in regions associated with social cognition, automatically commits crimes (Schleim, 2025; Mobbs et al., 2007). Contrary to what is often assumed, it is not a question of *whether* an act was caused or not, but rather the *kind* of causation (Greenberg and Bailey, 1994; Morse, 1994). Can the criminal act be attributed to a person or is it the result of a pathological disorder that exceeds the person's insight and control? Confusing the question of causation vs. non-causation with the question of legal responsibility occurs so frequently that Stephen J. Morse coined the “psycholegal error” (Morse, 1994). But criminal law is compatible with what we know of the causes of human action and it is thus unsurprising that the revolution in criminal law that some natural scientists have been calling for since the 19th century has not yet come about (Schleim, 2020b; Vincent, 2015). The debate on free will has often dealt with questions other than those of interest to criminal law.

Is it different in everyday life? The question is too complex to discuss in all detail here. But in line with Ryle's above-mentioned assumption and the structure of criminal law, it can be assumed that people in everyday life are also primarily concerned with the intent and responsibility of others: Let's assume that someone spills a glass of juice on somebody's favorite sweater. Then we want to know whether this person acted deliberately to harm someone. Or was it perhaps an accident? And if so, could the person have avoided the damage if they had been paying more attention? Or did they perhaps slip because of an uneven floor that they could not see and for which they are not responsible? And does the person acknowledge the mistake and apologize, perhaps offering compensation for the damage? As we can see, all these important questions in our everyday lives have nothing to do with abstract free will, but with insight and conscious control: did the person know what they were doing and were they in control of themselves? With these thoughts in mind, we can reinterpret the frequently cited neuroscientific experiments.

## Free will experiments reconsidered

In both Benjamin Libet's famous experiments from the 1980s and its variant in the fMRI scanner, experimental subjects were asked to perform a spontaneous movement: either move their hand or press a button (Libet et al., 1983a; Soon et al., 2008). These experiments were often interpreted to mean that the subsequent decision to move was determined by an unconscious brain process. To make this distinction, subjects had to specify the time of their conscious decision (for the movement). The measured neuronal processes before this moment were then labeled as unconscious.

In line with the previous sections, however, this is not the decisive question. Benjamin Libet, who did not deny the possibility of free will, also repeatedly pointed out that the “unconscious” neuronal activity he measured, the readiness potential, only stands for the *initiation* of a movement (Libet, 1985). As he and his colleagues reported at the time and other researchers later confirmed, this initiation does not necessarily lead to the execution of the action (Libet et al., 1983b; Trevena and Miller, 2010; Tretter et al., 2026). With regard to the example above, someone could feel the desire to pour a glass of juice over another person's sweater—but then *not* do it. In the much-cited original work by Libet et al. (1983a), it was already discussed that there must be a further process that leads from the initiation of the movement to its execution—and that nothing can yet be said about whether this process is conscious or unconscious.

Other aspects also fit with the interpretation that such experiments involve conscious control tasks: for example, the subjects were asked to consciously observe the dynamics of their psychological processes in order to determine the timing of the decision. In the fMRI study, increased activation was also found in regions such as the prefrontal cortex and precuneus, which are associated with executive control and conscious processes (Soon et al., 2008). In fact, a more recent follow-up study by this research group using a brain-computer interface showed that the execution of a movement can be halted in response to a consciously perceived stop signal up to 200 ms before the movement (Schultze-Kraft et al., 2016). Libet had already made a similar prediction (Libet, 2004). Accordingly, the discussion of free will should focus less on determinism and more on the possibilities and limits of conscious control (Schleim, 2024; Wittmann et al., 2025), in line with legal and everyday practice. By contrast, a real problem for our view of responsibility would occur if we are forced by unconscious brain activation to act in ways that we cannot consciously object to. This in turn would bring to mind the pathological excuses of law mentioned in the previous section.

## Summary and outlook

The free will debate still receives a lot of attention (Figure 1). In this opinion article, I have pointed out that this discussion has been going on with similar arguments since the 19th century. The physicist, philosopher and intellectual father of logical positivism Ernst Mach (1838–1916) suggested that a question that cannot be answered is probably wrongly formulated (Mach, 1903). The confusion I suspect in the free will debate consists in arguing endlessly about unprovable (in-) determinism, while in legal and social practice it is about responsibility—and subsequently also guilt. I therefore suggest that we look more closely at the possibilities and limits of conscious control. Improving scientific models of our decision-making should also advance the debate (Hechtlinger et al., 2026).

## Footnotes

1 I am grateful for the reviewer's critical remark that while I criticized the absence of definitions of “the will” in the free will debate, I didn't define “control” in an earlier version of this manuscript. The otherwise conceptually abundant *Philosophical Foundations of Neuroscience* mentions the term and its variations 94 times, but doesn't provide a definition, only examples like “A fully voluntary movement is one which the agent controls in its inception, continuation and termination” (Bennett and Hacker, 2022, p. 241). In a classic source, MacKay (1966) pointed out the conceptual origins of cognitive science in communication science and used the example of a thermostat which controls (regulates) the room temperature within a given range. For further discussion of control in relation to, for example, conflict monitoring and consciousness, the reader is referred to highly-cited sources on these questions (Botvinick et al., 2001; Suhler and Churchland, 2009).
